# Lived Experiences of Caregivers of Patients with Borderline Personality Disorder: A Phenomenological Study

**DOI:** 10.30476/IJCBNM.2020.83358.1154

**Published:** 2020-04

**Authors:** Ali Meshkinyazd, Abbas Heydari, Mohammadreza Fayyazi Bordbar

**Affiliations:** 1 Department of Nursing, School of Nursing and Midwifery, Mashhad University of Medical Sciences, Mashhad, Iran; 2 Nursing and Midwifery Care Research Center, School of Nursing and Midwifery, Mashhad University of Medical Sciences, Mashhad, Iran; 3 Department of Psychiatry, School of Medicine, Mashhad University of Medical Sciences, Mashhad, Iran

**Keywords:** Borderline personality disorder, Caregivers, Iran, Qualitative research

## Abstract

**Background:**

Having a patient with borderline personality disorder (BPD) in the family is a complicated and stressful experience. The caregivers’ experiences and the problems they have in care of patient with BPD have remained unknown. The aim of this research was to explore the experiences of the caregivers while living with BPD patients in Iran.

**Methods:**

This interpretive phenomenological research was performed on 10 caregivers of patients with BPD at Ibn-sina Hospital in Mashhad, Iran, in 2019. Purposeful sampling was used for sampling. Data were collected through semi-structured interviews and saturated after 16 interviews. The analysis of data was concurrently carried out using the method proposed by Diekelman (1989). The MAXQDA software (Ver.10) was used for data organization.

**Results:**

The participants in this study were aged 25 to 55 years. After data analysis, three themes (“life in hell”, “chain to the feet”, and “black shadow of stigma”) and six sub-themes (“disrupted from the life”, “self-discrepancy”, “care bottlenecks”, “in the fence of restriction”, “society dagger” and “resort to secrecy”) emerged.

**Conclusion:**

The results of this study showed that the caregivers of patients with BPD during the period of care were faced with a variety of problems. It is suggested that health policy-makers should pay more attention to the problems related to the mental health of caregivers.

## INTRODUCTION

Personality disorder is characterized by pervasive maladaptation and inflexibility in behavior, cognition, emotional state (excitement), and impulse control, that significantly deviates from one’s cultural expectations and often results in mental distress, social and occupational dysfunction. ^1^

Among the personality disorders, Borderline Personality Disorder (BPD) has the highest prevalence. ^2^ Diagnostic and Statistical Manual of Mental Disorders (DSM-5) estimates that the prevalence of this disorder in the community is about 1.6% and can increase to 5.9%. The prevalence rate of this disorder in primary health care centers is about 6%, in those who refer to outpatient mental health clinics, about 13%, and in patients admitted to mental hospitals, about 23%. ^3^ Suicide and self-harm are indicators of this disorder. 70% to 75% of these patients have a history of at least one self injury. These range from minor scratches, knocking the head on the wall, burning with cigarettes, up to high doses of drugs, and self-laceration with a knife. ^4^

Since, one of the goals of the World Health Organization in promoting mental health and the treatment of mental illnesses is to reduce the length of hospital stay and expand social services, the role of families in caring for these patients becomes even more important. ^5^

Caregivers of BPD, while being able to manage and control the behaviors of patients, play a vital role in maintenance and rehabilitation of patients. ^6^ Thus, caregivers suffer from severe physical, psychological and social pressures during patient care and control. ^7^ In fact, patients and their families are constantly affected by the changes resulting from the disorder and its treatment. These changes gradually reduce the level of performance and the ability of family members, destruct of emotional system and communication structures of family, affect the relationships among members, emergence of financial and economic problems, reduce social interactions of the family, change in roles, reduce life expectancy, and emerge the symptoms such as anger, feeling guilty and grief. ^8^

Overall, psychological burden of care, while reducing the quality of life of caregivers, can jeopardize their physical and mental health, ultimately leading to poor care, leaving the treatment or violent behavior with patients; these problems can exacerbate the patients’ disorder. ^9^

These caregivers show a high level of mental and physical distress compared to the normal population. ^10^ In this regard, one study showed that caregivers of BPD patients experienced more sadness than those of mentally ill patients. ^11^ In general, caregivers have many needs; nurses can identify and prioritize these needs by planning and using the nursing process. Supporting caregivers are the duty of all members of the care team, but nurses in this case are in a special position and in fact are the main supporters of family members in the hospital. ^12^

Although studies have been done on the treatment and care of these patients, unfortunately, what is often forgotten about helping these patients are the experiences and needs of the caregivers. Moreover, most of the studies were quantitative. For example, in one study, it was reported that after using the questionnaire, the respondents claimed that the comprehension of some of the words were difficult and that response options were often inadequate to describe their views. ^13^

A deeper understanding of these unknown aspects can facilitate the planning of a comprehensive health care plan. A full understanding of the caregivers’ experiences is not possible without a qualitative research. In the present study, the researchers aimed to use interpretive phenomenology because they aimed to gain a better understanding of the caregivers’ experience of their patient. Phenomenology is the lived experience of humans from the world of their everyday lives. In fact, it is a person’s lived experience that tells him/her what is real and true in his/her life. These lived experiences give meaning to a person’s perception of a particular phenomenon and are influenced by all internal and external factors. ^14^ Interpretive phenomenology focuses on personal experiences and meanings; in this research method the participants speak through language. However, they actually present their psychological world with what they say, and how they perceive their living conditions. ^15^ Therefore, the aim of this study was to explore the experiences of caregivers of patients with BPD by using an interpretive phenomenological approach. 

## MATERIALS AND METHODS

This study was a qualitative research with a hermeneutic phenomenological approach. The participants of the study were selected from caregivers of patients with BPD referring to Ibne-sina hospital in Mashhad. The inclusion criteria were at least 25 years of age, willingness to participate and interview, no history of known psychiatric disorders, and ability to share the experiences. Exclusion criteria include requests for abandonment and unwillingness to participate. Sampling continued until data saturation. Data collection was stopped when saturation occurred. That is, when no new data or themes were found in the new data collected. Purposeful sampling with maximum variation was done. Data collection through semi-structured interviews performed. The time and place of the interviews were determined by the priority chosen by the participants, most participants agreed to be interviewed in a quiet room at Ibn Sina Hospital and several interviews were also conducted at the participants’ homes, all of which being conducted by the researcher in a quiet location. The interviews lasted between 60 to 100 minutes. Interviews were recorded with the permission of the participants, and after each interview all conversations were written on the paper by the researcher.

Every interview was done by a PhD student nursing education. He introduced himself at the beginning of the interviews and then described the research aims and the interview steps to the participants. Then, he asked them if they were willing to participate, and then they gave their consent by signing the consent form. Then, the initial questions were asked. For example, “What comes to your mind when told about caring of patients with BPD?” and “What is your understanding of caring for a patient with BPD?” Then, probing questions were asked such as: “Can you explain more about this”, “How do you feel about them”, or “Can you clarify what you mean by the example”. After each interview, the audio file was converted into Microsoft word, and MAXQDA 10 software was used to better manage the data.

In the present study, data analysis was performed according to the proposed method of Diekelmann, Allen, and Tanner (1989). This method is one of the most widely used interpretive analysis methods in nursing research. In this method, the hermeneutic cycle is considered at all stages. Also, the discussion of the group that is included in the whole process of analysis provides more accurate data. ^16^ This method is a seven-stage process based on Heidegger’s phenomenology and the steps are as follows:

1- Reading all the interviews and texts in order to gain a general understanding, 2- Writing interpretive summary for each interview, 3- analysis of selected texts of interviews and identification and extraction performing group of the themes, 4- Returning to interviewers or participants to explain, clarify, and classify the cases of disagreement and inconsistencies in interpretations presented and writing a comprehensive analysis, 5- Comparing and contrasting the texts (interviews) with the aim of identifying, and describing common meanings, 6- Identifying and extracting fundamental patterns that link the themes and connect them, and 7- Providing a draft copy of the themes, along with selected transcripts of the interviews to individuals familiar with the method and content of the work. Their comments were included in the final text. ^17^

At this point, meaning units were obtained after several reading of texts. Then, common meanings and sub-themes were formed based on the similarity of the extracted meaning units. Finally, three themes were emerged. 

In order to achieve rigor of the study, we considered and used the criteria proposed by Lincoln and Guba in 1985, i.e. credibility dependability, confirmability and transferability. ^18^ Therefore, with emphasis on the selection of appropriate areas, information sources and eligible participants, close, accurate, and long-term partnerships, interactions, and adopting a team approach using the collective views of the research team, and re-referral to the participants, we ensured the validity and accuracy of the study.

Ethical consideration was observed through explaining the aims and methods used in the research for the participants, obtaining informed written consent from the research participants, confidentiality of all material presented, explaining the purpose of using the audio recording, remembering the voluntary participation in research, and the possibility of withdrawal at any stage of research. This study was approved by the local research ethics committee of Mashhad University of Medical Sciences (IR.MUMS. REC.1396.407). 

## RESULTS

The participants in this study were 10 caregivers of BPD with a mean age of 38.7±6.5 years. The participants’ demographic data are presented in Table 1.

**Table 1:** Demographic characteristics of the study participants

Participant’s No	Education	Gender	Interview of time (Minutes)	Duration of care (Year)
1	Primary school	Male	80	8
2	Primary school	Female	70	5
3	Diploma	Female	100	3
4	BSc	Male	90	12
5	MSc	Female	70	10
6	Primary school	Female	60	4
7	Diploma	Male	85	6
8	BSc	Male	75	9
9	MSc	Female	60	7
10	Diploma	Female	85	11

The data analysis led to the development of three themes and six sub-themes. The developed themes and sub-themes are listed in Table 2.

**Table 2:** Meaning units, Common meanings, Sub-themes, and Themes

Meaning units	Common meanings	Sub-themes	Themes
Inability in patient control	Chaos in life	Disrupted from the life	Life in hell
Inability in the management of life			
Feeling depressed	Negative feelings In life		
Feeling of failure in life			
Feeling of hate of patient			
Feeling regret about the patient			
Feeling angry towards the patient			
Wish the death of the patient			
Lack of enjoyment in life	Dark life		
Lack of comfort in everyday life			
Desire to die			
Blame yourself for a patient ‘s illness	Self blame for patient education	Self-discrepancy	
Feeling guilty in treating with patient			
Not having time to deal with physical problems	Neglect of yourself		
Physical discomfort			
Premature aging			
Disregard for individual health			
Fatigue in care	Overwhelming care	Care bottlenecks	Chain to the feet
Inability in care			
Confusion in care			
Pay high treatment costs	Economical pressure		
Ignore other members’ fees			
Inability to afford daily living expenses			
Insufficient insurance coverage			
Workplace stress	Social restrictions	In the fence of Restriction	
Educational problems for family members			
Restrictions on exercise			
Loss of marriage opportunities for family members			
Continuous care	Restrictions on daily life		
Disturbance in marietal relations			
Inability to carry out responsibilities			
Discomfort in response to the stigma of hospital staff	Inner turmoil to the stigma of others	Society dagger	Black shadow of stigma
Feeling heartbroken in response to the stigma of neighbors			
A sense of helplessness in responding to the stigma of close friends			
Loss of previous position in the view of relatives	Weakening of family status among relatives and acquaintances		
Family rejection by others			
Decreased communication with relatives			
Neighbors’ disgust of the family			
Ethical problems, Barrier for family Interactions			
Awareness of others from the illness of the spouse, barrier to communication	Concealment of disease	Resort to Secrecy	
Changing the behavior of others with awareness of disorder			
Not telling to others about disorder, for not understanding			
Maintain relationships with others by concealing disorder			
Secrecy, a way to avoid negative judgment			
Hospitalization,the factor that changes the view of others	Hiding Hospitalization		
Forced to hide hospitalization to prevent relatives’ curiosity			

### *Theme 1: Life in Hell*

1-a: Disrupted from the life: This sub-theme includes three common meanings: chaos in life, negative feelings in life and dark life. As caregivers begin to recognize the problems, endless sorrow for caregivers begins and spreads throughout the life. They were very sad during the conversation, and their sadness was evident on their faces. The caregivers knew their lives were far from the ideal. There was no happiness in their lives, and some of them wished to die.

In the present study, the lack of control in patient’s behavior was one of the factors that caused chaos in caregiver’s life. The patient’s behaviors were aggressive and the family was unable to control them (Table 3).

**Table 3:** Quotation, Meaning units, Common meanings for “chaos in life”

Quotation	Meaning units	Common meanings
“I can’t stand it anymore; there is no other way in my mind. I don’t know what to do anymore. How should I control it? My brain is blocked. Where should I start from? I do not know”.	Inability in patient control	chaos in life
“In this life, I’m going slightly mad. I have to be mindful of everything and nothing is in its place. On the one hand, i have to be mindful of the issues of life, and on the other hand, I have to deal with my daughter’s abnormal behavior. I desire to go to a place where no one is seen and I want to be alone”.	Inability in the management of life	

Living with patients leads to negative emotions including feeling of failure in life, depression, hate of patient, regret about the patient, and desire for the death of the patient.

One of the caregivers said: *“I feel caught up in the swamp and I’m trying to save myself. I am constantly crying as if my emotions were dripping with crying and giving me some peace”* (participant number 1).

Another caregiver said: *“This patient creates great problems for the family and we avoid him. Although our child lives with us, I do not have any sense to him. We hate him”* (participant number 9).

The caregivers of these patients do not enjoy the pleasures of life and perhaps we can say that nothing is good for them in their lives.

In this regard, one of the caregivers said: *“With the disaster that i have experienced in life, I have no interest in life anymore. I think so involved in the problems that I am not happy about anything. I do not enjoy my life. Most of the time, I feel sad , and I don’t have a good day in my life”* (participant number 3).

Most of the participants wanted to end their lives and preferred to die. 

One of the caregivers said: *“When I die, I feel comfortable; I’m tired of this life. I have a hard life. When I sleep at night, I do not want to wake up in the morning. I do not like this life. I love to die”* (participant number 1).

1-b: Self-discrepancy: Another sub-theme was self-discrepancy that includes two common meanings: self-blame in patient education and neglect of oneself. Caregivers are blamed for the disorder and feel guilty about dealing with their patient.

One of them stated: *“In my child’s illness, I don’t consider myself as innocent. If I had paid more attention to him and had not engaged myself too much, this son wouldn’t have been like this. I did take him to recreational places .I bought a tablet for him to have fun and he had nothing to do with us. I did not love him.”* (participant number 1).

Participants in this study, despite the experience of physical problems in the limbs and body organs, were not paying enough attention to pursuing and maintaining their health. In the process of illness and treatment, caregivers neglect themselves and sacrifice their life.

In this regard, one of the caregivers said: *“I cry when I am alone and I’m very upset for her; I had nerve problems; for example, I had chest pain yesterday, as if my chest had been punctured. I feel that something is hitting my chest, and I feel I’m dying. Before, I didn’t have that kind of pain, but I do not care much about these pains because all of my thoughts are about my daughter. I say only that he will be better, now I can endure these problems”* (participant number 7).

### *Theme 2: Chain to the Feet*

2-a: Care bottlenecks: It was difficult for the applicants to manage jobs and day-to-day care, so they felt that care bottlenecks could make them tired and overwhelmed, and this sub-theme included imposing disease costs on the care-givers and lacking financial support from insurance agencies. The care-givers claimed that the disorder imposed high costs on them. This sub-theme contains two common meanings: overwhelming care and economical pressure.

One of the main concerns of the majority of participants was using all their time and energy to care the patient and become tired and powerless. Problems with caring for participant 5 were so painful that he was crying and said: *“Taking care of her is so hard 24 hours a day; I have to be careful, and I must be careful not to do anything dangerous. I have become really tired and helpless”*.

Another caregiver said: *“Some days, I get up and feel bored, generally weak and fatigued, so that I cannot do anything else; excessive fatigue does not allow my daily activities to be done”* (participant number 9). 

The participants expressed their concerns about the financial problems and economic burdens of the disease that had affected their lives. 

One other caregiver said: *“We are not in a good, i.e. economical situation in our lives; we have problems with our normal living expenses. Sometimes, we cannot even meet the cost of our food.”* (participant number 3).

Another caregiver said: *“My husband’s doctor told me to get new foreign medicine for our patient. These medicines are expensive; I am anxious about how to provide it. Preparation of these drugs has caused a financial problem in the family ”*( participant number 10).

As to insufficient insurance coverage, one caregiver stated: *“There is no good insurance for BPD patients. Many drugs are not covered by insurance agency.”*( participant number 2).

2-b: In the fence of restriction

This sub-theme contains two common meanings: social restriction and restrictions on daily life. Full-time patient care prevented caregivers from pursuing other life activities and they neglected other roles and duties toward themselves and their families such as disregarding other members and impaired marital relationships; all their attention was paid to their patients, so they could not handle other events around them. Of all the caregivers, only one person had mentioned success in performing other roles besides caring. Patient care has been associated with social constraints and limitations in daily life.

In this regard one of the participants said: *“There is no celebration at our house. At least for the past two years, we had not had any trips together”* (participant number 8).

Another caregiver said: *“I can’t do my maternal and spouse duties well. I get caught by this patient. I can’t properly handle the responsibilities of life”* (participant number 6).

### *Theme 3: Black Shadow of Stigma*

The main theme of *“Black Shadow of stigma”* reflects the negative beliefs of the community about clients with psychiatric disorders. Many people often readily accept this idea and prejudice about psychiatric disorders and treat everyone with mental illness in this notorious group. This leads to inappropriate and discriminatory behavioral reactions to them, which has devastating effects on their patient and caregivers. This theme contains two subthemes: society dagger and resort to secrecy.

3-a: Society dagger: 

This sub-theme includes the negative views of the society towards BPD; the negative belief towards BPD patients causes the family’s position to be weakened and rejected by relatives and acquaintances. Also, when their friends, acquaintances and neighbors stigmatized their patients, caregivers felt frustrated, uncomfortable, and heartbroken. This sub-theme contains two common meanings: inner turmoil to the stigma of others and weakening of family status among the relatives and acquaintances.

One caregiver said: *“The neighbors were telling their child that crazy guy is dangerous. He is caught in the curse of God; the devil has penetrated his body. When I heard that, my heart was breaking, I was disappointed and felt humiliated ”* (participant number 5).

Another participant said: *“I was already versatile for relatives and acquaintances. They all came to me to solve their problems, but since my son has been ill, my relatives are no longer the same as before. They no longer care about us, and they don’t even invite us to parties”* (participant number 2).

3-b: Resort to secrecy

The sub-theme of *“resort to secrecy”* in the participants’ experiences indicated that some caregivers hided the disease to get rid of the others’ judgments and their negative views. Therefore, as a protective strategy, they attempted to hide the disorder and hospitalization of the patient from others. They were concerned that if they introduce their child or spouse as a person with a psychiatric disorder, they would be judged and treated differently. They were trying to control the world around them by concealing their sickness.

One of the caregivers said: *“I didn’t want others to know that my husband had a mental illness. If I tell them, they will have a different look at my husband and definitely change their behavior.”* (participant number 4)

Another caregiver said: *“My relatives have a negative view of mental illness. When my husband was in the mental health hospital, our relatives wanted to see him, but if they came, they would call him crazy. When he was discharged from the mental health hospital, they told him *“you are crazy and have to be hospitalized at a mental hospital.”* Hence, when my husband was in a mental hospital, I didn’t tell anyone”* (participant number 3).

## DISCUSSION

The purpose of this study was to explore the experiences of caregivers with BPD patients in Iran. After data analysis, three themes (“life in hell”, “chain to the feet”, and “black shadow of stigma”) and six sub-themes (“disrupted from the life”, “self-discrepancy”, “care bottlenecks”, “in the fence of restriction”, “society dagger” and “resort to secrecy”) emerged.

The findings of this study have shown that patients with BPD change the caregivers’ living conditions. As caregivers of this study described, their lives were very difficult because of the uncontrollable and annoying behaviors of the patient that puts them in a stressful situations. The lives of the caregivers of BPD patients have been of interest to some researchers. Results of some studies revealed that disability and suffering from illness and a stressful life for caregivers changed their health and quality of life. ^19 , 20^ In this regard, another study concluded that the stress of caring for a family member with a mental illness decreases their quality of life. ^21^ In the present study, due to the inability to control the patients and their living conditions, caregivers had family disturbances and experienced low quality of life.

In the current study, the majority of participants reported feelings of sadness, suffering, hopelessness, and helplessness in their experiences. They were psychologically distressed and their physical health was threatened. In general, the presence of a person with psychiatric disorders leads to a crisis in the family. Living with a mentally ill person causes the family members to be under severe stress, and affects all aspects of their lives. Since a family member becomes mentally ill, it disrupts the vital balance of the family system in relationships, roles, and desires. Therefore, the integrity of the family system cannot be ignored in the occurrence of such disorders. ^22^ Severe stressors such as violent and destructive behaviors and alteration of past lifestyle can put the caregivers and other family members in crisis. These problems can expose the family members to physical and emotional problems. ^23^ Patients’ behavioral problems, including abnormal unpredictable and aggressive behaviors are challenges for caregivers that lead to confusion in patient control. ^24^ In the present study, the major concerns of the family are the malicious behaviors of the patient and the way to control and manage these behaviors, and often these behaviors make the care-givers frustrated.

Consistent with a previous study, caregivers in the current study stated that following the patient care process, they also experienced mental health problems, emotional dysfunction and mood changes including depression, anxiety, and fatigue. ^25^ Previous studies have investigated the psychiatric symptoms in care-givers quantitatively and paid less attention to the impact of these symptoms on the caregivers’ performance. ^19 , 20^ However, in the present study, the effects of these symptoms on the care-givers’ performance were also revealed and explained. Also, the participants reported that psychological and emotional problems such as feeling angry, depressed or failure, lack of motivation and hopelessness were a barrier to performing their functional aspects, which were consistent with the results of previous studies. ^26 , 27^

In this study, the participants complained of problems with their physical health such as physical discomfort, premature aging and fatigue associated with the care of BPD patients. Their experiences also indicated that some of the physical disorders were caused by emotional distress. They often attributed these problems to psychosocial stress. A study showed that care-givers of mentally ill patients experience various physical disabilities and some chronic illnesses such as diabetes, chest pain and heart disease. ^28^ In another study, muscle tension and pain, physical exhaustion and lack of sleep are common experiences of the care-givers who endanger their health. ^29^ These studies are consistent with our study.

Care-givers in this study experienced that there was a lack of balance between their areas of work performance and they could not pay attention to all of their functional areas and ignored some issues. Patient care responsibilities make the care-givers neglect their functional areas. In this regard, one study showed that living with a person with a psychiatric illness and caring for them can affect all areas of the caregiver’s life and function; ^30^ also, another study showed that caregivers devoted a great deal of time to patient care and this interferes with all functional areas and activities of their lives. ^31^

In the current study, care-givers reported that caring for BPD patients created problems in their occupational activities. One of the most important problems for care-givers was the lack of focus during work causing workplace stress. In this regard, one research revealed that care-givers were constantly distressed and worried about their patients and these concerns persisted while they were at work and prevented them from focusing on work. ^32^

Caring for a BPD patients causes the care-givers to leave their social activities. One previous study have also confirmed the decline in social and communication activities. ^33^ Consistent with the findings of the present study, lack of time is another obstacle for the care-givers to engage in social and communication activities. ^26^ The results of this study show that the care-givers have to reduce or quit their recreational activities in order to spend more time and energy for the patient, and care for the patient is an obstacle to performing these activities. Consistent with the findings of the present study, one research showed that one of the negative consequences of caring was not paying for recreational activities. ^31^ In another study conducted on care-givers, most family members spent their time to caring for the patient and never had the time to enjoy life. ^34^

Most care-givers in this study complained of high costs and lack of adequate insurance coverage. They also had problems with their daily living expenses, which was consistent with previous studies. ^35 , 36^ Another problem for caregivers is disruption of their daily activities. Caregivers said that their daily life had been affected by the patient’s needs and care, so that they could not perform their daily activities. In this regard, one study showed that caregivers endured a lot of stress and it affected all activities of their daily life. ^37^ In Iran, these caregivers confront with more severe financial problems. Thus, it seems that the development of mental health disorders such as BPD through imposing heavy costs can reduce the comfort of life of the caregivers and limit their daily living activities. These findings highlight that the authorities should consider the financial problems associated with this disorder and the impact of this pressure on caregivers.

Another major problem in caregivers is the socio-cultural wrong beliefs about BPD patient in the community. The caregivers in the present study were worried about the negative beliefs in the society and felt shame and hopelessness as a result of stigmatizing the patient and family, thus keeping the disease as a secret. This finding was consistent with previous study. ^38^ Caregivers are reluctant to report illness because the label of the illness affects the view of others about the patient and caregiver. Consistent with this finding, the study showed that caregivers believed that community attitudes toward psychiatric disorders were negative and because in society there are these negative beliefs, carergivers are obliged to hide the disorder of the family member. ^39^ In the present study, caregivers also concealed their hospitalization of patients to prevent ridicule and harassment by others. Consistent with this finding, a study showed that hospitalization in a psychiatric hospital was kept secret by the patients’ families. ^40^

This study had some limitations and strengths. The caregivers had a rich experience of the BPD patient and eagerly explained their experiences. Because of the lack of research in this area, the current study is one of the first research to demonstrate this phenomenon in Iran. According to other qualitative studies, the generalizability of this study is limited. In addition, this research may not include all experiences of the caregivers who are living in other cities.

## CONCLUSION

Results of this study showed how the experiences of BPD caregivers faced with a variety of unpredictable problems in the care of the patients. Because of the care pressure, caregivers have a low quality of life. Two key elements in relation to these caregivers should never be forgotten. First, every member of society should respect and pay attention to the vital role of caregivers of BPD patients, and this can reduce a lot of stress these caregivers experience. Second, authorities and policymakers can consider the problems of these caregivers by development of appropriate legislation that facilitates the way to solve their problems. Therefore, the need for comprehensive support seems to be essential for caregivers of patients with BPD.

## References

[1] Bateman A, Campbell C, Luyten P, Fonagy P ( 2018). A mentalization-based approach to common factors in the treatment of borderline personality disorder. Current Opinion in Psychology.

[2] Hong V ( 2016). Borderline personality disorder in the emergency department: Good psychiatric management. Harvard Review of Psychiatry.

[3] Hepper EG, Hart CM, Meek R ( 2014). Narcissism and empathy in young offenders and non-offenders. European Journal of Personality.

[4] Clarkin JF, Meehan KB, Lenzenweger MF ( 2015). Emerging approaches to the conceptualization and treatment of personality disorder. Canadian Psychology/Psychologie Canadienne.

[5] Akbari M, Alavi M, Irajpour A, Maghsoudi J ( 2018). Challenges of family caregivers of patients with mental disorders in Iran: A narrative review. Iranian Journal of Nursing and Midwifery Research.

[6] Fossati A, Somma A ( 2018). Improving family functioning to (hopefully) improve treatment efficacy of borderline personality disorder: An opportunity not to dismiss. Psychopathology.

[7] Bulbena-Cabré A, Perez-Rodriguez MM, Porges S ( 2017). Understanding Anxiety in Borderline Personality Disorder. Current Treatment Options in Psychiatry.

[8] Cackowski S, Neubauer T, Kleindienst N ( 2016). The impact of posttraumatic stress disorder on the symptomatology of borderline personality disorder. Borderline Personality Disorder and Emotion Dysregulation.

[9] Zeng Y, Zhou Y, Lin J ( 2016). Perceived burden and quality of life in Chinese caregivers of people with serious mental illness: A path analysis. International Journal of Psychosocial Rehabilitation.

[10] Dorozenko KP, Ridley S, Martin R, Mahboub L ( 2016). A journey of embedding mental health lived experience in social work education. Social Work Education.

[11] Grenyer BFS, Bailey RC, Lewis KL ( 2019). A randomized controlled trial of group psychoeducation for carers of persons with borderline personality disorder. Journal of Personality Disorders.

[12] Alipoor T, Abedi HA, Masoudi R ( 2016). Care experiences and challenges of inpatients’ companions in Iran’s health care context: A qualitative study. International Journal of Medical Research and Health Sciences.

[13] Bailey RC, Grenyer BF ( 2013). Burden and support needs of carers of persons with borderline personality disorder: a systematic review. Harvard Review of Psychiatry.

[14] VanScoy A, Evenstad SB ( 2015). Interpretative phenomenological analysis for LIS research. Journal of Documentation.

[15] Iared VG, de Oliveira HT, Payne PG ( 2016). The aesthetic experience of nature and hermeneutic phenomenology. Journal of Environmental Education.

[16] Crowther S, Ironside P, Spence D, Smythe L ( 2017). Crafting stories in hermeneutic phenomenology research: a methodological device. Qualitative Health Research.

[17] Manookian A, Dehghan Nayeri N, Negarandeh R, Shali M ( 2015). The lived experiences of intensive care patients on transfer to a general ward. Nursing Practice Today.

[18] Lincoln YS, Guba EG (1985). Naturalistic inquiry.

[19] Sharma N, Chakrabarti S, Grover S ( 2016). Gender differences in caregiving among family-caregivers of people with mental illnesses. World Journal of Psychiatry.

[20] Nogueira DJ, Minamisava R, Teles SA ( 2019). Factors Associated with Marital Satisfaction and Quality of Life in Family Caregivers of Patients with Mental Disorders. International Journal of Environmental Research and Public Health.

[21] Hayes L, Hawthorne G, Farhall J ( 2015). Quality of life and social with schizophrenia: policy and outcomes. Community Mental Health Journal.

[22] Mottaghipour Y, Tabatabaee M ( 2019). Family and Patient Psychoeducation for Severe Mental Disorder in Iran: A Review. Iranian Journal of Psychiatry.

[23] Carbonell Marqués Á, Navarro-Pérez JJ ( 2019). The care crisis in Spain: an analysis of the family care situation in mental health from a professional psychosocial perspective. Social Work in Mental Health.

[24] Kaur A, Mahajan S, Deepti SS, Singh T ( 2018). Assessment of role of burden in caregivers of substance abusers: a study done at Swami Vivekananda Drug De-addiction Centre, Govt. Medical College, Amritsar. International Journal of Community Medicine and Public Health.

[25] Garcia-Williams AG, McGee RE ( 2016). Responding to a suicidal friend or family member: a qualitative study of college students. Death Studies.

[26] Ohara C, Komaki G, Yamagata Z ( 2016). Factors associated with caregiving burden and mental health conditions in caregivers of patients with anorexia nervosa in Japan. Biopsychosocial Medicine.

[27] Bekuma L, Ababaw M, Melluish S ( 2018). Psychological Distress, Subjective Burden and Stigma among Caregivers of People with Mental Illness in Gondar University Hospital, Ethiopia. Ethiopian Renaissance Journal of Social Sciences and the Humanities.

[28] Kim G, Allen RS, Wang SY (2018). The relation between multiple informal caregiving roles and subjective physical and mental health status among older adults:
Do racial/ethnic differences exist?. The Gerontologist.

[29] Buus N, Caspersen J, Hansen R ( 2014). Experiences of parents whose sons or daughters have (had) attempted suicide. Journal of Advanced Nursing.

[30] Polo-López R, Salaberria K, Echeburúa E ( 2015). Effectiveness of a psychological support program for relatives of people with mental disorders compared to a control group: a randomized controlled trial. Behaviour Research and Therapy.

[31] Alzahrani SH, Fallata EO, Alabdulwahab MA ( 2017). Assessment of the burden on caregivers of patients with mental disorders in Jeddah, Saudi Arabia. BMC Psychiatry.

[32] Pearce J, Jovev M, Hulbert C ( 2017). Evaluation of a psychoeducational group intervention for family and friends of youth with borderline personality disorder. Borderline Personality Disorder and Emotion Dysregulation.

[33] Karimirad MR, Seyedfatemi N, Noghani F ( 2018). Resiliency family caregivers of people with mental disorders in Tehran. Iranian Journal of Nursing Research.

[34] Ahmad Ramli FZ, Tilse C, Wilson J ( 2017). Qualitative interviewing of Malay caregivers: stigma and mental health problems of older adults. International Journal of Culture and Mental Health.

[35] Vaingankar JA, Chong SA, Abdin E ( 2016). Care participation and burden among informal caregivers of older adults with care needs and associations with dementia. International Psychogeriatrics.

[36] Marimbe BD, Cowan F, Kajawu L ( 2016). Perceived burden of care and reported coping strategies and needs for family caregivers of people with mental disorders in Zimbabwe. African Journal of Disability.

[37] Jagannathan A, Thirthalli J, Hamza A ( 2014). Predictors of family caregiver burden in schizophrenia: Study from an in-patient tertiary care hospital in India. Asian Journal of Psychiatry.

[38] Park S, Park KS ( 2014). Family stigma: A concept analysis. Asian Nursing Research.

[39] Vaghee S, Kashani Lotfabadi M, Salarhaji A ( 2018). Effect of Psychology Internship on Stigma of Psychiatric Disorders in Nursing Students. Journal of Medical Education.

[40] Muralidharan A, Lucksted A, Medoff D ( 2016). Stigma: a unique source of distress for family members of individuals with mental illness. The Journal of Behavioral Health Services & Research.

